# Neuropsychiatric Symptoms in Multiple Sclerosis: A Case Report

**DOI:** 10.1192/j.eurpsy.2025.1073

**Published:** 2025-08-26

**Authors:** A. Picallo Vieito, S. Buyo Lagares, M. Pardal Iglesias, M. Grueiro Cao, A. Lagoa Pego, C. Ramil López, C. Fernández Ovies, A. Parada Barcia, P. Piñeiro Magro, L. González Pereira, J. Ricoy Chaín, R. Prieto Perez

**Affiliations:** 1 Psychiatry, Hospital Universitario A Coruña, A Coruña, Spain

## Abstract

**Introduction:**

The relationship between bipolar disorder (BD) and multiple sclerosis (MS) has gained increasing attention due to the complex interplay between neurological and psychiatric symptoms in affected individuals. BD can significantly impair quality of life, and its prevalence in MS patients remains a critical area of investigation.

We present the case of a a 42 year-old woman. She was admitted to the Psychiatric Hospitalization Unit due to a manic episode with psychotic symptoms. Her psychiatric history includes a visit to the Mental Health Unit one year prior for anxiety-depressive symptoms. Notably, she has a diagnosis of Multiple Sclerosis from 2022.

**Objectives:**

Based on the described clinical case, we aim to review the available literature on the relationship between bipolar disorder and multiple sclerosis.

**Methods:**

A systematic review of the literature was conducted using databases such as PubMed, Scopus, and PsycINFO. Studies published up to 2023 that reported on the prevalence and clinical characteristics of BD in MS patients were included. Data were extracted and synthesized to provide a comprehensive overview.

**Results:**

The review identified 20 relevant studies, with pooled data indicating a prevalence of BD in MS patients ranging from 5% to 20%. Key factors influencing prevalence included demographic variables, such as age and sex, as well as the presence of other psychiatric comorbidities. The literature suggests that inflammatory processes and neurobiological changes in MS may contribute to the development of mood disorders, including BD.

**Conclusions:**

There is significant evidence linking bipolar disorder with multiple sclerosis, highlighting the importance of comprehensive mental health assessments in MS patients. Future research should focus on understanding the pathophysiological mechanisms behind this association and developing tailored interventions to improve overall patient outcomes.

**Disclosure of Interest:**

None Declared

